# CA-125 Early Dynamics to Predict Overall Survival in Women with Newly Diagnosed Advanced Ovarian Cancer Based on Meta-Analysis Data

**DOI:** 10.3390/cancers15061823

**Published:** 2023-03-17

**Authors:** Eleni Karamouza, Rosalind M. Glasspool, Caroline Kelly, Liz-Anne Lewsley, Karen Carty, Gunnar B. Kristensen, Josee-Lyne Ethier, Tatsuo Kagimura, Nozomu Yanaihara, Sabrina Chiara Cecere, Benoit You, Ingrid A. Boere, Eric Pujade-Lauraine, Isabelle Ray-Coquard, Cécile Proust-Lima, Xavier Paoletti

**Affiliations:** 1 Gustave Roussy, Office of Biostatistics and Epidemiology, Université Paris-Saclay, 94805 Villejuif, France; 2 Oncostat, Labeled Ligue Contre le Cancer, CESP U1018, Inserm, Université Paris-Saclay, 94805 Villejuif, France; 3 Beatson West of Scotland Cancer Centre, NHS Greater Glasgow and Clyde, Glasgow G12 0XH, UK; 4 Cancer Research UK Clinical Trials Unit, Institute of Cancer Sciences, University of Glasgow, Glasgow G12 0YN, UK; 5 Department of Gynecologic Oncology, Institute for Cancer Genetics and Informatics, Oslo University Hospital, 0424 Oslo, Norway; 6 Department of Medical Oncology, Cancer Centre of Southeastern Ontario, Queen’s University, Kingston, ON K7L 3N6, Canada; 7 Foundation for Biomedical Research and Innocation, Translational Research Center for Medical Innovation, Kobe 650-0047, Japan; 8 School of Medicine, The Jikei University, Tokyo 105-8461, Japan; 9 Department of Urology and Gynecology, Istituto Nazionale Tumori IRCCS Fondazione G. Pascale, 80131 Napoli, Italy; 10 EMR UCBL/HCL 3738, Faculté de Médecine Lyon-Sud, Université Lyon, Université Claude Bernard Lyon 1, 69100 Lyon, France; 11 Medical Oncology, Institut de Cancérologie des Hospices Civils de Lyon (IC-HCL), CITOHL, Centre Hospitalier Lyon-Sud, GINECO, GINEGEPS, 69495 Lyon, France; 12 Department of Medical Oncology, Erasmus MC Cancer Institute, 3015 GD Rotterdam, The Netherlands; 13 ARCAGY-GINECO, GINECO, GINEGEPS, 75008 Paris, France; 14 Centre Léon Bérard, GINECO, 69008 Lyon, France; 15 UMR1219, Bordeaux Population Health Research Center, Inserm, University of Bordeaux, 33000 Bordeaux, France; 16 Faculty of Medicine, University of Versailles Saint-Quentin, Université Paris Saclay, 78000 Versailles, France; 17 INSERM U900, Statistics for Personalized Medicine, Institut Curie, 92210 Saint-Cloud, France

**Keywords:** ovarian cancer, CA-125, biomarker, linear mixed model, meta-analysis

## Abstract

**Simple Summary:**

Cancer antigen 125 (CA-125) is a protein found at a high concentration in the blood of patients with specific types of cancer, mainly ovarian cancer. In 2004, the Gynecologic Cancer Intergroup (GCIG) proposed criteria defining response to treatment, as well as disease progression, based on the CA-125 concentration. Ever since, for the follow-up of ovarian cancer patients, the CA-125 concentration and/or CT-scans are used. This paper aims to compare different summaries of CA-125 evolution in the 3 to 6 months following treatment initiation in newly diagnosed advanced ovarian cancer and explore their prognostic capacity to predict overall survival. Based on individual patient data from the GCIG meta-analysis, we propose the most appropriate timeframe between follow-up and the prediction horizon in order to obtain robust, dynamic, individual predictions.

**Abstract:**

(1) Background: Cancer antigen 125 (CA-125) is a protein produced by ovarian cancer cells that is used for patients’ monitoring. However, the best ways to analyze its decline and prognostic role are poorly quantified. (2) Methods: We leveraged individual patient data from the Gynecologic Cancer Intergroup (GCIG) meta-analysis (N = 5573) to compare different approaches summarizing the early trajectory of CA-125 before the prediction time (called the landmark time) at 3 or 6 months after treatment initiation in order to predict overall survival. These summaries included observed and estimated measures obtained by a linear mixed model (LMM). Their performances were evaluated by 10-fold cross-validation with the Brier score and the area under the ROC (AUC). (3) Results: The estimated value and the last observed value at 3 months were the best measures used to predict overall survival, with an AUC of 0.75 CI 95% [0.70; 0.80] at 24 and 36 months and 0.74 [0.69; 0.80] and 0.75 [0.69; 0.80] at 48 months, respectively, considering that CA-125 over 6 months did not improve the AUC, with 0.74 [0.68; 0.78] at 24 months and 0.71 [0.65; 0.76] at 36 and 48 months. (4) Conclusions: A 3-month surveillance provided reliable individual information on overall survival until 48 months for patients receiving first-line chemotherapy.

## 1. Introduction

Ovarian cancer (OC) is the seventh most common cause of cancer mortality in women worldwide, with a survival rate of 46% at 5 years after diagnosis [1]. First-line treatment consists of primary or interval debulking surgery and platinum- and taxane-based chemotherapy, which may be combined with maintenance treatments including bevacizumab or poly (ADP-ribose) polymerase (PARP) inhibitors, if applicable. Recently, PARP inhibitors have demonstrated very promising gains in progression free survival (PFS) and overall survival (OS), especially for patients with BRCA-mutated ovarian cancer [2,3,4,5]. Cancer antigen 125 (CA-125) is a marker mainly related to ovarian cancer, but it may also be elevated in other conditions. According to the Gynecologic Cancer Intergroup (GCIG) criteria, it is used to monitor the patient’s response to treatment in cases of recurrent disease or to define progression after first-line therapy [6,7]. Although in 2010, the routine measurement of CA-125 was discouraged [7,8], two strategies for the follow-up of women after primary treatment are now used: (i) clinical follow-up with CA-125 and imaging on clinical indication or (ii) CT-scans throughout follow-up and optional CA-125 testing [9]. However, the contribution of CA-125 is debated, as clinical trials implement systematic CA-125 and CT-scan surveillance, especially in maintenance treatment settings. The best ways to analyze CA-125 decrease, its timeframe and reproducibility across trials, and the risk of error are insufficiently documented.

Assessing the prognostic value of the early evolution of CA-125 for time-to-event endpoints raises statistical challenges. The CA-125 trajectory can be summarized in multiple ways (e.g., CA-125 value at baseline, KELIM at 3 months, CA-125 value, or decrease at the prediction time (e.g., 3 and 6 months)), and its prognostic role has been studied in many isolated studies [10,11,12,13,14]. Nevertheless, the performance of these methods has never been evaluated in an individual patient data meta-analysis. CA-125 is a biomarker prone to measurement error and biological variation (i.e., some values may appear higher or lower than anticipated from the overall trajectory), so that raw summaries of observed CA-125 may be suboptimal for assessing CA-125 prognostic value. Furthermore, CA-125 may not be available at a particular timepoint, and an imputation method may be necessary [15]. Therefore, some authors have proposed reliance on modeling techniques that handle noisy and sparsely measured biomarkers [16,17] to better assess the evolution of CA-125. Recently, the use of the CA-125 ELIMination Rate Constant K (KELIM) [18], which is based on longitudinal CA-125 measures as well as pharmacokinetic and pharmacodynamic parameters of treatment, has gained important interest in multiple settings (in recurrent disease, for neo-adjuvant treatments, and for initial treatments). In the adjuvant and neo-adjuvant settings, the prognostic role of the KELIM at 100 days (i.e., 3 months) has been tested in several trials [19,20]. Alternatively, statistical techniques such as mixed-effect modeling have been successfully developed in the case of prostate cancer to model the prostate-specific antigen dynamic over time [17,21]. A recent study proposed a CA-125 rate estimate that is easy to quantify in order to aid in decision making regarding second-line treatment for patients with recurrent high-grade serous ovarian cancer [22]. These statistical methods for longitudinal data are thus central to the assessment of the prognostic value of the trajectory estimated over various timeframes. Considering timepoints of less than three months could be challenging due to the scarcity of CA-125 measurements in this timeframe. Conversely, longer periods of monitoring of CA-125, such as six months, may improve the model’s accuracy and the discriminatory value of the CA-125 decline. This may entail a CA-125 collection burden, which may lead to an elevated number of follow-up visits.

When studying the prediction of a time-to-event endpoint based on a prone-to-error biomarker, where the timeframe differs between the longitudinal process (a few months) and the survival endpoints (a few years), a landmark approach may be an appropriate approach. Briefly, its principle is to set up a landmark time from which the prediction is to be performed and restrict the population to event-free subjects at this timepoint. Summaries of the dynamics of the biomarker before the landmark time serve to predict progression or death after the landmark time [23,24].

When developing prediction tools, the assessment of the predictive performance needs to be carried out carefully. The value of the marker for predicting the risk of death can be measured using the Brier score and time-dependent area under the ROC curve (AUC) with estimators adapted to the time-to-event context [25,26,27]. The predictive performance may be overoptimistic when assessed on the same data used for training the model. To correct this bias, external validation or cross-validation techniques can be used [28].

This work aims to assess the performance of CA-125 early dynamics and determine the most appropriate landmark timepoint (i.e., the timeframe required to enrich the statistical prediction models) so as to predict OS at the best future timepoint in newly diagnosed patients with advanced ovarian cancer treated with taxane- and platinum-based chemotherapy. We used individual patient data (IPD) meta-analysis from the GCIG meta-analysis group study, which has a large and diverse sample size, permitting a cross-validation of the results between patients and studies as well as between subgroups based on the patients’ characteristics.

## 2. Materials and Methods

This report follows the Preferred Reporting Items for Systematic Reviews and Meta-analyses (PRISMA)—IPD guidelines and the Transparent reporting of a multivariable prediction model for individual prognosis or diagnosis (TRIPOD) statement for the registration of the protocol, trial identification, data collection and integrity, assessment of bias, and sensitivity analyses [29,30]. This meta-analysis was registered with PROSPERO (CRD42017068135). The Ethics Committee of Gustave Roussy Cancer Center, Villejuif, France, approved this study, and the French Data Protection Authority waived the need for informed consent due to the use of deidentified data.

### 2.1. Study Population

To develop and validate a statistical predictive model of the dynamics of CA-125 in relation to OS, we used the IPD of the GCIG meta-analysis. This included patients with newly diagnosed ovarian cancer of the International Federation of Gynecology and Obstetrics FIGO stages IC to IV whose data were collected from randomized controlled trials published from January 2001 to September 2016 (GCIG meta-analysis of ovarian cancer [31]). From the initial set of 17 trials, we selected those that investigated initial systemic treatments after surgery (no maintenance treatments).

For the present analysis, only trials that collected serum CA-125 levels at baseline (before the start of the treatment) and repeatedly during follow-up were selected. Data collection followed the original research protocol. To be eligible, every patient had to have at least two CA-125 measures, including one at baseline, and complete information regarding overall survival and progression.

Data checking was performed to ensure the data quality. Levels of the biomarker greater than 15,000 were considered as outliers, and peri-operative CA-125 measures were excluded, as they are affected by the surgical intervention.

### 2.2. Outcomes

The primary endpoint was OS, defined as the time from randomization to death of any cause. Patients alive at the cut-off date were censored at the last follow-up date.

The prognostic value of the levels of CA-125 at 3 and 6 months from randomization and the rate of CA-125 decline were evaluated.

The model was developed based on patients treated with either the ‘standard chemotherapy’ (Paclitaxel + Carboplatin and Epirubicin/Doxorubicin) or the same chemotherapy with an investigational treatment, as none of the included trials showed significant differences in treatment effect between the two arms. A sensitivity analysis, after excluding the patients from the investigational arms, was carried out.

The following patient characteristics were available at baseline: age, performance status, FIGO stage, histological subtype and grade, and residual disease after surgery (≥1 cm, <1 cm, unknown).

### 2.3. Statistical Methods

In order to study the biomarker–OS association over time, a landmark analysis was performed, and different post-baseline times, noted as s, were considered [19]. All the available data up to s were modeled to predict the risk of death from time s to time s + t, with t denoting the horizon. The investigated prognostic factors based on the early CA-125 trajectory included (i) the observed CA-125 value at the landmark time, where if the former was missing, the closest previous measure was retained (OVLT), and (ii) the observed relative decline (ORD) in the biomarker between baseline and the last CA-125 measure before the landmark. In addition, three CA-125 summaries estimated using a hierarchical linear mixed-effect model were considered: (iii) the estimated value at the landmark time (EVLT) and (iv–v) the estimated slopes at baseline (ESB) and at the landmark time (ESLT).

For these three summaries, repeated measures of the log-transformed CA-125 were analyzed using a hierarchical linear mixed model (HLMM) [32], which handles the hierarchical structure of the data, the inherent measurement error, and missing values according to the missing-at-random mechanism. Three hierarchical levels were distinguished: (i) the between-trial variability, using random effects at the trial level, (ii) the within-patient correlation, using patient-specific random effects, and (iii) the variability due to observation-specific measurement errors.

To account for the nonlinear dynamics of log-CA-125 over time, we considered a basis of natural cubic splines over time with 3 or 4 internal knots for the landmark time at 3 or 6 months, respectively [33]. The knots were placed at the beginning of the follow-up (i.e., 0.5, 1, and 2 or 3 months for 3 and 4 knots, respectively) in order to capture possible rapid declines in the biomarker, and external knots were placed at the 2%- and 98%-percentiles of the measurement times. Knot selection was based on the Akaike criteria (AIC) of the model. For each patient, we included random effects on each natural cubic spline function to capture the patient-specific CA-125 deviation to the mean trajectory. For each study, a random intercept was further used to capture the trial-specific deviation.

The summaries were derived from the HLMM using the Best Linear Unbiased Predictor for the trial- and patient-specific random effects.

### 2.4. Evaluation of the Predictive Performance of the CA-125 Summaries

The summaries were included as fixed effects in a Cox proportional hazards model considering study-specific baseline hazards. Heterogeneity across studies of the associations between the summaries and survival was tested with a likelihood ratio test that compared the partial likelihood of the stratified model with the partial likelihood of the stratified model using trial specific summary effects [34,35]. 

**Figure 1:**
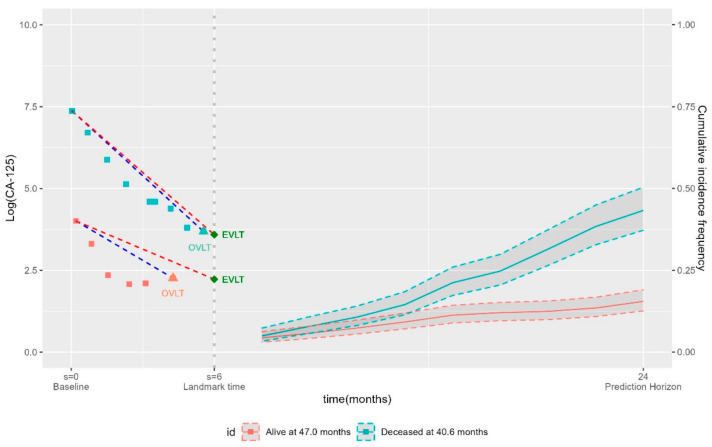
Illustration of the landmark framework. CA-125 trajectories of observed values up to the landmark time of s = 6 months for two patients at risk of death at the landmark time (#1 alive after 56.9 months, and #2 deceased after 24.5 months). Their overall survival was assessed at t = 24 months. Summaries of the early CA-125 trajectory were obtained from values up to the landmark time (the blue dashed lines represent the observed slope, and the red dashed lines represent the estimated slope).

As shown in Figure 1, the probability of death was predicted from the landmark time s at the horizon time s + t according to the CA-125 summary computed at time s, with s = 3 and 6 months and t = 24, 36, 48, and 60 months in a subset of studies with a sufficient follow-up.

The predictive performance of these predictions was assessed using the AUC and Brier score in order to determine the most appropriate landmark time and to explore the robustness for various prediction horizon times. To account for censored times-to-event between the time s and the time s + t, estimators of these two quantities were weighted by the inverse probability of censoring (IPCW) [36]. Both measures were used to assess the quality of the prediction tool. The AUC can be seen as a concordance measure between patients with high and low risks of death [37], while the Brier score measures calibration and discrimination. Both measures range between 0 and 1. An AUC below 0.7 suggests a moderate discriminatory performance.

The calibration was assessed graphically: patients were split into groups defined by quantiles of the predicted event probabilities, and the results were plotted against the observed risk of death.

To correct for the over-optimistic performance obtained on the training datasets, we applied a 10-fold cross-validation technique on the patient level. The original dataset was partitioned into 10 sub-samples of the same size. For each sub-sample, the predictions were computed using the model trained on the 9 remaining sub-samples. The predictions from the 10 sub-samples were then pooled for the performance assessment. The average and standard deviation of the Brier score and the AUC over 50 replicates of the cross-validation technique were finally reported to account for fluctuations in the partitions. The corresponding 95% interval of the bootstrap distribution was calculated.

To further investigate the added value of a summary of CA-125 dynamics, we finally compared the Cox model stratified by study and adjusted for the evaluated summary to either (i) the null model, i.e., a survival model stratified by study that served as a reference, or (ii) the same Cox model with the summary of the dynamics and with or without CA-125 level at baseline.

In a secondary analysis, the prognostic value of CA-125 was evaluated based on subgroups of patients defined by their baseline characteristics, such as the FIGO stage and residual disease.

Finally, for the known cutoff of 35 for the CA-125 value, we evaluated the positive predictive value (PPV) and the negative predictive value (NPV) at the analyzed time horizons.

The statistical analyses were performed using R (version 4.1.2, R foundation for Statistical Computing, Vienna, Austria), with R package RiskRegression for the predictive performances assessment and the lme4 package for the HLMM.

## 3. Results

### 3.1. Study Selection and Characteristics

Among the 17 trials in the GCIG meta-analysis, repeated CA-125 measures were available for 13 trials. Fours trials that investigated maintenance treatments were further excluded. A total of nine trials were then selected, with four and five trials, respectively, investigating new initial treatments and the intensification of an initial treatment, leading to a total sample of 5573 patients.

**Scheme 1:**
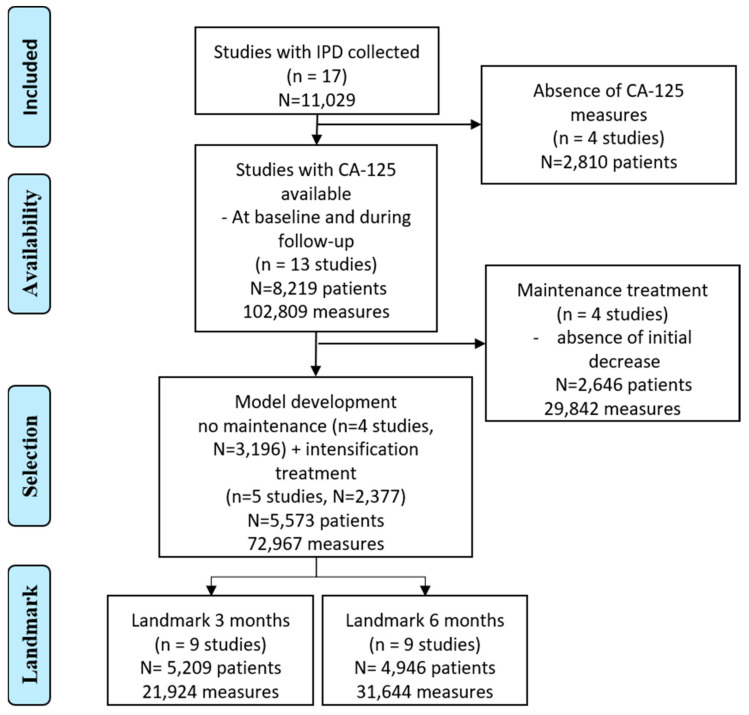
Flowchart: study selection.

More than 72,967 CA-125 measures were collected (see the flowchart in Scheme 1). The median number of CA-125 measures per patient was 11 (IQR [7,8,9,10,11,12,13,14,15,16,17]), with a range between 2 and 100 values, as described in the Supplementary Materials, Table S1. The trials and patient characteristics are described in Table 1 and Table 2, respectively and in the Supplementary Materials, Table S1. A total of 2570 deaths were reported, and the median OS was 4.11 years [3.94–4.33].

### 3.2. Endpoint and Landmark Timeframe

We computed survival at the time horizons of 24, 36, 48, and 60 months for two landmark times, s = 3 and 6 months. A total of 5209 and 4946 patients were alive at these two landmark times, respectively, and included in the analysis set.

### 3.3. Performance of CA-125 Summaries

**Figure 2:**
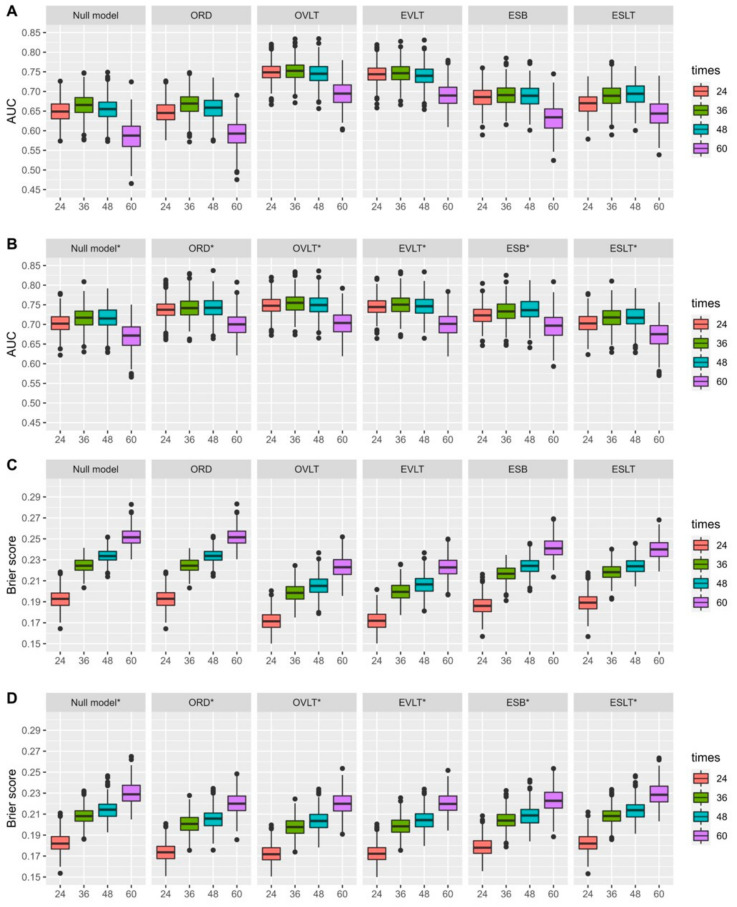
The 10-fold cross-validated AUCs (**A**,**B**) and Brier scores (**C**,**D**) for the prediction of overall survival at 24, 36, 48, and 60 months from CA-125 history up to 3 months, considering different CA-125 summaries (ORD, OVLT, EVLT, ESB, ESLT) adjusted (**B**,**D**) or not (**A**,**C**) based on the CA-125 value at baseline. Null model: survival model stratified by study, ORD: observed relative decline, OVLT: observed value at landmark time, EVLT: estimated value at landmark time, ESB: estimated slope at baseline, ESLT: estimated slope at landmark time. * models adjusted for CA-125 at baseline. Note: The SCOTROC-1 trial was excluded for the landmark time at 6 months and prediction time at 36 months and for the landmark time at 3 months and prediction time at 48 months. The SCOTROC-4 and MITO-7 trials were excluded for both landmark times and for the prediction time of 60 months.

**Figure 3:**
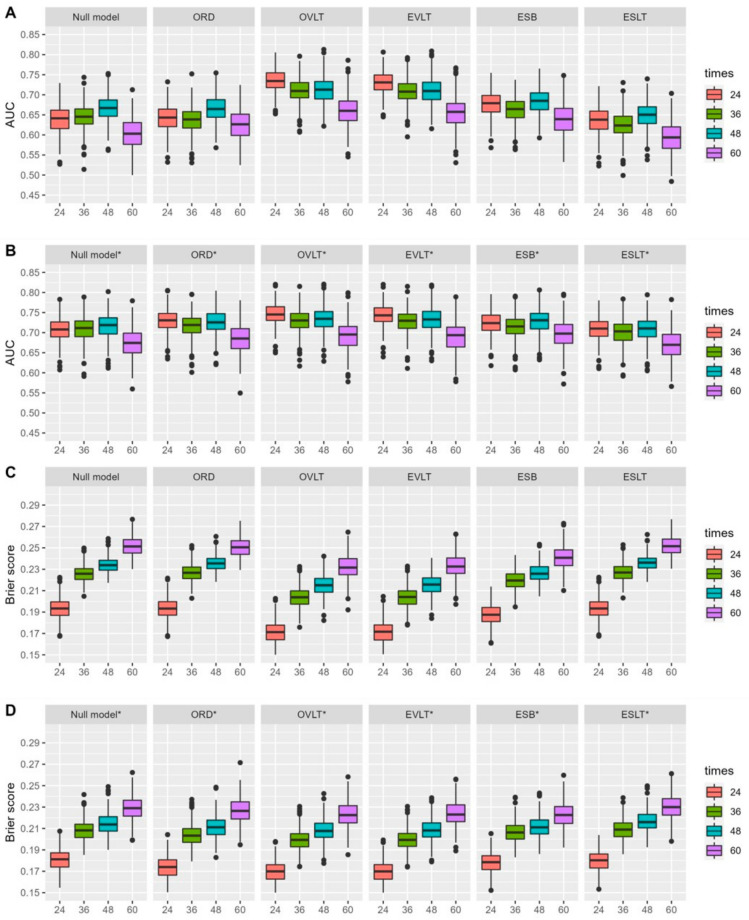
The 10-fold cross-validated AUCs (**A**,**B**) and Brier scores (**C**,**D**) for the prediction of overall survival at 24, 36, 48, and 60 months from CA-125 history up to 6 months, considering different CA-125 summaries (ORD, OVLT, EVLT, ESB, ESLT) adjusted (**B**,**D**) or not (**A**,**C**) based on the CA-125 value at baseline. Null model: survival model stratified on study, ORD: observed relative decline, OVLT: observed value at landmark time. Null model: survival model stratified by study, ORD: observed relative decline, OVLT: observed value at landmark time, EVLT: estimated value at landmark time, ESB: estimated slope at baseline, ESLT: estimated slope at landmark time. * models adjusted for CA-125 at baseline. Note: The SCOTROC-1 trial was excluded for the landmark time at 6 months and prediction time at 36 months and for the landmark time at 3 months and prediction time at 48 months. The SCOTROC-4 and MITO-7 trials were excluded for both landmark times and for the prediction time of 60 months.

The cross-validated performances of the various CA-125 summaries are reported in Figure 2 and Figure 3, and further results are described in the Supplementary Materials, Tables S2 and S3. Regardless of the summary in question, the AUC were relatively similar at the 24-, 36-, and 48-month horizon times, with a drop in performance at 60 months. In contrast, the shorter the prediction horizon was, the smaller the error in the prediction was, as measured by the Brier score. The model had a good calibration, as shown in Supplementary Materials Figures S1–S5.

#### 3.3.1. Landmark at 3 Months

The estimated value at 3 months and the last observed value before or at 3 months provided the best predictive performances at all the horizon times with, for instance, AUCs of 0.745 IC 95% [0.699, 0.796] and 0.749 [0.702, 0.796], respectively, and statistically significant heterogeneity across trials for both summaries. These AUCs can be compared with the AUC of the null model (only accounting for the trial effect), which was 0.648 [0.593; 0.702] at the 24-month horizon time (Figure 2, Supplementary Materials Table S2).

The estimated slope showed a modest predictive accuracy. For instance, at 24 months, the estimated slope at baseline, without adding the CA-125 value at baseline, provided an AUC of 0.685 [0.637; 0.739], with significant heterogeneity across trials (*p* = 0.0009). The estimated slope and ORD at 3 months showed AUCs of 0.668 [0.616; 0.722] (with heterogeneity (*p* = 0.005)) and 0.646 [0.590, 0.697] (with non-significant heterogeneity across trials (*p* = 0.102)), respectively.

At the horizon time of 24 months, the prognostic impact of CA-125 at baseline (with a single variable included in the model stratified by trial) was 0.703 [0.659, 0.752]. After adding the CA-125 value at baseline, the predictive performances of the estimated and observed CA-125 values at the landmark time remained virtually the same as those without the CA-125 value at baseline (0.745 [0.701, 0.794] and 0.748 [0.705, 0.796], respectively). However, the estimated slope and ORD at 3 months, together with CA-125 at baseline, showed improved predictive performances at all times with, for instance, AUCs of 0.703 [0.658–0.752] and 0.738 [0.693, 0.789] at 24 months.

Of note, the AUC of 0.75 indicates that for two random patients, there is a 75% probability that the patient with the lowest CA-125 value at 3 months will have the longest survival when restricting to the window between 3 and 24 months.

#### 3.3.2. Landmark at 6 Months

Six-month landmark analyses performed in a manner similar to the 3-month analyses, with a lower performance for a longer prediction horizon time. The estimated value at 6 months and the last observed value before 6 months had AUCs of 0.731 [0.678; 0.778] and 0.735 [0.683; 0.783], respectively, at 24 months and AUCs of 0.656 [0.586; 0.734] and 0.66 [0.59; 0.734] at 60 months (Figure 3, Supplementary Materials Table S3).

#### 3.3.3. CA-125 Summary Performance for Subgroups of Patients Based on Characteristics

In the case of the FIGO stage III patients, the estimated value at 3 months provided an AUC value of 0.709 [0.640; 0.772] and a Brier score of 0.190 [0.167, 0.214] vs. 0.684 [0.446, 0.878] and a Brier score of 0.09 [0.062; 0.123] for FIGO IC and II at 24 months. For residual disease ≥1 cm vs. <1 cm at 24 months, the AUCs, without adding CA-125 at baseline, were 0.707 [0.634; 0.768] and 0.687 [0.589; 0.790], respectively. Note that the model performed equivalently after adding CA-125 baseline value (see Supplementary Materials Tables S4 and S5 and Figures S6 and S7).

#### 3.3.4. Sensitivity Analysis and Predictive Values of the CA-125 Normal Range

The sensitivity analysis of the patients from the standard treatment arm showed equivalent results for both landmark times. (Supplementary Materials, Tables S6 and S7 and Figures S8 and S9).

Finally, we examined the sensitivity and specificity of the CA-125 value at 3 months. A patient with a CA-125 value < 35 had a 20% probability of death at 24 months, and 30%, 38%, and 66% at 36, 48, and 60 months, respectively. On the contrary, a patient with a CA-125 value > 35 had a 51% probability of death at 24 months, and 66%, 75%, and 79% at 36, 48, and 60 months, respectively.

## 4. Discussion

In patients with newly diagnosed FIGO stage II to IV ovarian cancer, the serum CA-125 level at 3 months after the end of primary treatment showed the best prediction of the overall survival probability at 24, 36, and 48 months compared to various measures of the CA-125 decrease rate. The last observed CA-125 value before or at 3 months and the estimate based on the repeated-measures model were associated with AUCs at 24 months of 0.749 and 0.745, respectively. A landmark time of 6 months did not improve the predictive performances, suggesting that early assessment provides the best trade-off between clinical application and statistical performance. The predictive capacity of both CA-125 summary measures remained equivalent after adding the CA-125 baseline value. Important improvement in the rate measures was observed after adding the CA-125 baseline value; for example, the observed rate of decline reached 0.738 at 24 months vs. 0.646 without the addition of the CA-125 baseline value. This reflects the importance of considering the CA-125 value at baseline in order to better interpret CA-125 kinetics. Despite the presence of heterogeneity, probably due to different inclusion criteria or healthcare standards between studies, the IPD meta-analytic context and the cross-validation allowed for robust results to be drawn.

A value of CA-125 above 35 at 3 months led to a higher risk of death, such as 51% at 24 months or up to 79% at 60 months.

In the case of 3-month surveillance, CA-125 kinetics could help in decision making regarding the intensification of the follow-up or the anticipation of a treatment change. More precisely, a patient with a CA-125 level concentration above 35 at 3 months would benefit from closer monitoring, with the measurement of the CA-125 concentration and CT scans in order to anticipate a potential need for treatment change. These patients may be good candidates for clinical trials upon progression.

Predictions at over 60 months post-landmark should be interpreted carefully, since all the measures performed poorly compared to the earlier prediction times. A lack of follow-up could explain such a performance. The main limitation is that recently approved drugs such as PARP-inhibitors were not included in this meta-analysis, since the trials were published between 2001 and 2016. Although the standard is still the chemotherapy backbone, maintenance treatment including PARP-inhibitors is now the standard of care. This may lead to different associations between CA-125 and OS. An update including trials with newly approved drugs, such as PARP-inhibitors, would be of interest to further quantify the prognostic ability of CA-125 in this context. Furthermore, we relied on linear mixed models to overcome the problems of missing and sparse data. Although we considered flexible functions of time and carefully assessed the goodness-of-fit, this approach is still based on parametric assumptions.

Recently, the subgroup of patients with HRD- or BRCA-positive status have gained great interest as a separate subtype and may lead us to question the robustness of our results for this subgroup [38]. This matter could be investigated through an update of the meta-analysis with the most recent agents. Finally, this meta-analysis is limited to serum CA-125, although other biomarkers with potential prognostic capacities, such as human epididymis protein type 4 (HE4), osteopontin, mesothelin (MSLN), and folate receptor α (FOLR1), can also be measured depending on the circumstances and state of the patients [39]. As a result, we concentrated our research on serum CA-125, which is routinely used in practice, and tried to quantify its prognostic capacity. Nevertheless, the same type of work could be extended, combining other markers in specific contexts to assess the risk of progression or death.

The assessment of KELIM in patients treated with maintenance veliparib and those treated with maintenance bevacizumab showed promising results that suggested a similar performance between KELIM and the reported chemotherapy agents. Nevertheless, further assessment of the predictive value of CA-125 at 3 months in the setting of PARP maintenance is required.

The development of model-based approaches has been motivated by the impact of the variability between different assays used in routine practice on the interpretation of outcomes of the one- and two-timepoint strategies. Interestingly, the 3-month time window found in the present study is consistent with the 100-first-treatment-day period used for KELIM calculation.

## 5. Conclusions

To conclude, the surveillance of CA-125 at 3 months after the initiation of treatment could help to provide individual information for patients based on the initial CA-125 trajectory. More precisely, this could help to provide an informative tool so as to guide clinicians in decision making regarding overall survival up to 48 months.

## Figures and Tables

**Table 1:** Description of included studies.

	Trial Name	Number of pts	Number of CA-125 Measures	Median Survival
Intensification therapy	total	2377	21,978	3.87 (3.6; 4.11)
	MITO-7	529	1429	NR
	GINECO-2007	109	634	2.63 (2.25; 3.39)
	TURBO-2014	253	3421	3.74 (3.23; 4.30)
	JGOG-3016	557	3475	6.72 (5.30; NA)
	SCOTROC-4	929	14,019	2.75 (2.54; 2.97)
No maintenance	total	3196	49,989	4.33 (4.08; 4.66)
	JGOG-3017	632	9403	NR
	NSGO-2012	774	9904	3.60 (3.21; 3.98)
	SCOTROC-1	1052	12,582	2.86 (2.73; NA)
	CCTG-OV.16	737	18,100	3.84 (3.49; 4.28)
Total		5573	72,967	4.11 (3.94; 4.33)

Abbreviations: NR, not reached.

**Table 2:** Patients’ characteristics by trial (N = 5209).

Trials	FIGO					Residual Disease			
	I	II	III	IV	.	<1 cm	≥1 cm	NA	.
MITO-7	41 (8.9%)	42 (9.1%)	273 (59.2%)	105 (22.8%)		NA	NA		
GINECO-2007	0	0	78 (80.4%)	19 (19.6%)	1	14 (14.4%)	83 (85.6%)		1
TURBO	0	19 (7.9%)	156 (65%)	65 (27.1%)		173 (72.7%	63 (26.5%))	2 (0.8%)	2
JGOG-3016	0	102 (18.6%)	364 (66.5%)	81 (14.8%)		258 (47.2%)	275 (50.3%)	14 (2.6%)	
SCOTROC-4	118 (13.9%)	75 (8.8%)	549 (64.6%)	108 (12.7%)		300 (35.3%)	550 (64.7%)		
JGOG-3017	395 (66.1%)	69 (11.5%)	112 (18.7%)	22 (3.7%)		548 (91.6%)	48 (8%)	2 (0.3%)	
NSGO-2012	0	94 (12.7%)	533 (71.8%)	115 (15.5%)		317 (42.7%)	425 (57.3%)		
SCOTROC-1	70 (7.2%)	127 (13%)	645 (65.9%)	137 (14%)		332 (33.9%)	647 (66.1%)		
CCTG-OV.16	0	65 (9.4%)	468 (67.4%)	161 (23.2%)		325 (57%)	238 (41.8%)	7 (1.2%)	124

Abbreviations: ‘.’: Missing information, NA: Not available.

## Data Availability

Individual patient data (IPD) were requested for each eligible trial for all randomized patients. All data were checked with a standard procedure which follows the recommendations of the Cochrane working group on meta-analysis using individual patient data. Each trial was analyzed individually, and the resulting survival analyses and data description were sent to the trialists for review.

## Supplementary Materials

The following supporting information can be downloaded at: https://www.mdpi.com/article/10.3390/cancers15061823/s1, Table S1: Description of patients’ characteristics (N = 5573). Table S2: 10-fold cross-validated AUC (A,B) and Brier score (C,D) for the prediction of overall survival at 24, 36, 48 and 60 months from CA125 history up to 3 months when considering different CA125 summaries. Table S3: 10-fold cross-validated AUC (A,B) and Brier score (C,D) for the prediction of overall survival at 24, 36, 48 and 60 months from CA125 history up to 6 months when considering different CA125 summaries (Landmark time 6 months). Table S4: Predictive performances by subgroups for estimated CA-125 value at 3 months. Table S5: Predictive performances by subgroups for estimated CA-125 value at 6 months. Table S6: 5-fold cross-validated AUC (A,B) and Brier score (C,D) for the prediction of overall survival at 24, 36, 48 and 60 months from CA125 history up to 3 months when considering different CA125 summaries. Half population including only Standard regimen arm. Table S7: 5-fold cross-validated AUC (A,B) and Brier score (C,D) for the prediction of overall survival at 24, 36, 48 and 60 months from CA125 history up to 6 months when considering different CA125 summaries. Half population including only Standard regimen arm. Figure S1: Calibration plot for the observed rate of decline (ORD)—3 months. Figure S2: Calibration plot for the observed value at landmark time (OVLT)—3 months. Figure S3: Calibration plot for the estimated value at landmark time (EVLT)—3 months. Figure S4: Calibration plot for the estimated slope at baseline (ESB) (for landmark 3 months). Figure S5: Calibration plot for the estimated slope at landmark time (ESLT)—3 months. Figure S6: Predictive performances measured by AUC (A) and Brier Score (B) by subgroups for estimated CA-125 value at 3 months. Figure S7: Predictive performances measured by AUC (A) and Brier Score (B) by subgroups for estimated CA-125 value at 6 months. Figure S8: 5-fold cross-validated AUC (A,B) and Brier score (C,D) for the prediction of overall survival at 24, 36, 48 and 60 months from CA125 history up to 3 months in Standard regimen arm when considering different CA125 summaries (ORD, OVLT, EVLT, ESB, ESLT) adjusted (B,D) or not (A,C) on the CA125 value at baseline. Figure S9: 5-fold cross-validated AUC (A,B) and Brier score (C,D) for the prediction of overall survival at 24, 36, 48 and 60 months from CA125 history up to 6 months in Standard regimen arm when considering different CA125 summaries (ORD, OVLT, EVLT, ESB, ESLT) adjusted (B,D) or not (A,C) on the CA125 value at baseline.
