# Supplementary material for: CA-125 Early Dynamics to Predict Overall Survival in Women with Newly Diagnosed Advanced Ovarian Cancer Based on Meta-Analysis Data
Source: Cancers (Basel). 2023 Mar 17;15(6):1823. doi: 10.3390/cancers15061823 (PMC10047009; doi:10.3390/cancers15061823)

Supplementary Materials

Table S1: Description of patients' characteristics (N=5573)

Variable		**Total (9 trials)**		**Population alive at 3 mois**		**Population alive at 6 mois**	
Age	n	5573		5209		4946	
	Mean (sd)	58.4 (10.8)		58.3 (10.8)		58.2 (10.6)	
	Median (Q1 ; Q3)	59 (51 ; 66)		58.3 (51 ; 66)		58.1 (51 ; 66)	
	Min ; Max	18 ; 88		18 ; 88		21 ; 88	
Performance status	n (m.d.)	5568 (5)		5204 (5)		4941 (5)	
	0	2740	49.2%	2597	49.9%	2523	51.1%
	1	2376	42.7%	2196	42.2%	2063	41.7%
	2+	452	8.1%	411	7.9%	355	7.2%
Figo	n (m.d.)	5572(1)		5208(1)		4945(1)	
	Stage I	657	11.8%	624	12%	625	12.6%
	Stage II	610	10.9%	593	11.4%	581	11.7%
	Stage III	3401	61%	3178	61%	2993	60.5%
	Stage IIV	904	16.2%	813	15.6%	746	15.1%

Histological subtype	n (m.d.)	5568 (5)		5204 (5)		4941 (5)	
	Serous carcinoma	2778	49.9%	2641	50.7%	2546	51.5%
	Endometrioid	504	9.1%	475	9.1%	461	9.3%
	Mucinous	154	2.8%	127	2.4%	113	2.3%
	Clear cell	881	15.8%	822	15.8%	787	15.9%
	Other	1111	20%	1009	19.4%	917	18.6%
	Unknown	140	2.5%	130	2.5%	117	2.4%
Histological grade	n (m.d.)	4275 (1298)		4019 (1190)		3813 (1133)	
	Well differentiated	451	10.5%	426	10.6%	408	10.7%
	Moderate differentiated	1112	26%	1051	26.2%	991	26%
	Poorly/Undifferentiated	2712	63.4%	2542	63.2%	2414	63.3%
Surgical procedure	n (m.d.)	5020(553)		4727 (482)		4450 (496)	
	Primary complete resection	4366	87%	4111	87%	3885	87.3%
	Delayed surgery	161	3.2%	144	3.0%	124	4.8%
	No surgery	493	9.8%	472	10%	441	9.9%
Maximal residual disease	n (m.d.)	4909(664)		4621 (588)		4350 (596)	
	<1cm	2356	48%	2267	49.1%	2209	50.8%
	>=1cm	2526	51.5%	2329	50.4%	2119	48.7%
	Unknown	27	0.6%	25	0.5%	22	0.5%

Assessment of progression	n	5573		5209		4946	
	CT-scan	632	11.3%	598	11.5%	598	12.1%
	CA-125-Clinical(confirmation with CT)	3712	66.6%	3483	66.9%	3247	65.6%
	Both	1229	22.1%	1128	21.7%	1101	22.3%
CA-125 at baseline	n	5573		5209		4946	
	Mean (sd)	687.50 (1552.73)		683.05 (1542.01)		653.64 (1481.97)	
	Median (Q1 ; Q3)	144 (44 ; 539.24)		142 (43 ; 532)		136 (41 ; 501)	
	Min ; Max	0.82 ; 14400		0.82 ; 14400		0.82 ; 14400	
log(CA-125 +1) at baseline	n	5573		5209		4946	
	Mean (sd)	5.12 (1.69)		5.11 (1.69)		5.06 (1.69)	
	Median (Q1 ; Q3)	4.98 (3.81 ; 6.29)		4.96 (3.78 ; 6.28)		4.92 (3.74 ; 6.22)	
	Min ; Max	0.60 ; 9.58		0.60 ; 9.58		0.60 ; 9.58	
number of repeated CA-125 measures per patient	n	5573		5209		4946	
	Mean (sd)	13.09		13.58		13.74	
	Median (Q1 ; Q3)	11 (7 ; 17)		12 (7 ; 17)		12 (7 ; 18)	
	Min ; Max	2 ; 100		2 ; 80		2 ; 80	

Table S2 : 10-fold cross-validated AUC (A,B) and Brier score (C,D) for the prediction of overall survival at 24, 36, 48 and 60 months from CA125 history up to 3 months when considering different CA125 summaries

A. AUC	Time	T=24	T=36	T=48	T=60
	Null model	0.648 [0.593 ; 0.702]	0.665 [0.608 ; 0.717]	0.654 [0.594 ; 0.711]	0.586 [0.51 ; 0.652]
	ORD	0.646 [0.59 ; 0.697]	0.668 [0.611 ; 0.724]	0.657 [0.599 ; 0.711]	0.591 [0.514 ; 0.659]
	OVLT	0.749 [0.702 ; 0.796]	0.751 [0.704 ; 0.801]	0.746 [0.69 ; 0.799]	0.695 [0.634 ; 0.753]
	EVLT	0.745 [0.699 ; 0.796]	0.746 [0.698 ; 0.794]	0.741 [0.688 ; 0.795]	0.691 [0.631 ; 0.75]
	ESB	0.685 [0.637 ; 0.739]	0.691 [0.635 ; 0.749]	0.689 [0.633 ; 0.745]	0.632 [0.572 ; 0.701]
	ESLT	0.668 [0.616 ; 0.722]	0.689 [0.635 ; 0.743]	0.694 [0.639 ; 0.748]	0.643 [0.583 ; 0.707]
B. AUC	Null model*	0.703 [0.658 ; 0.752]	0.717 [0.664 ; 0.771]	0.716 [0.663 ; 0.77]	0.669 [0.604 ; 0.725]
	ORD*	0.738 [0.693 ; 0.789]	0.742 [0.69 ; 0.791]	0.743 [0.689 ; 0.798]	0.7 [0.64 ; 0.763]
	OVLT*	0.749 [0.705 ; 0.796]	0.754 [0.704 ; 0.804]	0.75 [0.696 ; 0.801]	0.703 [0.644 ; 0.762]
	EVLT*	0.745 [0.701 ; 0.794]	0.75 [0.701 ; 0.799]	0.746 [0.691 ; 0.801]	0.7 [0.641 ; 0.756]
	ESB*	0.723 [0.677 ; 0.77]	0.734 [0.682 ; 0.787]	0.738 [0.68 ; 0.792]	0.696 [0.635 ; 0.752]
	ESLT*	0.704 [0.658 ; 0.752]	0.718 [0.665 ; 0.772]	0.719 [0.665 ; 0.773]	0.672 [0.608 ; 0.73]
C. Brier	Null Model	0.193 [0.174 ; 0.212	0.225 [0.212 ; 0.239]	0.234 [0.222 ; 0.247]	0.252 [0.236 ; 0.269]
	ORD	0.193 [0.174 ; 0.212]	0.225 [0.212 ; 0.239]	0.234 [0.222 ; 0.247]	0.252 [0.236 ; 0.269]
	OVLT	0.171 [0.153 ; 0.191]	0.199 [0.181 ; 0.216]	0.205 [0.188 ; 0.223]	0.223 [0.204 ; 0.243]
	EVLT	0.172 [0.153 ; 0.191]	0.2 [0.183 ; 0.217]	0.206 [0.189 ; 0.224]	0.223 [0.204 ; 0.242]
	ESB	0.186 [0.169 ; 0.206]	0.217 [0.202 ; 0.231]	0.224 [0.209 ; 0.239]	0.241 [0.225 ; 0.261]
	ESLT	0.189 [0.171 ; 0.209]	0.218 [0.204 ; 0.233]	0.224 [0.21 ; 0.238]	0.24 [0.224 ; 0.257]
D. Brier	Null model*	0.182 [0.164 ; 0.2]	0.208 [0.192 ; 0.225]	0.214 [0.198 ; 0.232]	0.23 [0.212 ; 0.252]
	ORD*	0.173 [0.154 ; 0.192]	0.201 [0.183 ; 0.218]	0.205 [0.186 ; 0.224]	0.22 [0.201 ; 0.24]
	OVLT*	0.172 [0.153 ; 0.19]	0.198 [0.18 ; 0.216]	0.204 [0.186 ; 0.223]	0.22 [0.202 ; 0.24]
	EVLT*	0.172 [0.153 ; 0.191]	0.199 [0.181 ; 0.216]	0.204 [0.186 ; 0.224]	0.22 [0.202 ; 0.24]
	ESB*	0.178 [0.16 ; 0.197]	0.204 [0.188 ; 0.221]	0.208 [0.19 ; 0.227]	0.223 [0.205 ; 0.244]
	ESLT*	0.182 [0.164 ; 0.201]	0.208 [0.192 ; 0.225]	0.214 [0.197 ; 0.231]	0.229 [0.212 ; 0.251]

Null model: survival model stratified on study, ORD: Observed relative decline, OVLT: Observed Value at landmark time, EVLT: Estimated value at landmark time, ESB: Estimated slope at baseline, ESLT: Estimated slope at landmark time

* models with CA-125 at baseline

Table S3: 10-fold cross-validated AUC (A,B) and Brier score (C,D) for the prediction of overall survival at 24, 36, 48 and 60 months from CA125 history up to 6 months when considering different CA125 summaries (Landmark time 6 months)

	Time	T=24	T=36	T=48	T=60
A. AUC	Null model	0.639 [0.574 ; 0.699]	0.646 [0.58 ; 0.704]	0.667 [0.606 ; 0.728]	0.603 [0.527 ; 0.672]
	ORD	0.643 [0.579 ; 0.703]	0.638 [0.575 ; 0.698]	0.665 [0.606 ; 0.726]	0.625 [0.556 ; 0.696]
	OVLT	0.735 [0.683 ; 0.783]	0.71 [0.653 ; 0.762]	0.712 [0.654 ; 0.765]	0.66 [0.59 ; 0.734]
	EVLT	0.731 [0.678 ; 0.778]	0.708 [0.648 ; 0.757]	0.709 [0.652 ; 0.762]	0.656 [0.586 ; 0.734]
	ESB	0.677 [0.615 ; 0.731]	0.663 [0.598 ; 0.72]	0.683 [0.618 ; 0.735]	0.639 [0.564 ; 0.706]
	ESLT	0.636 [0.574 ; 0.696]	0.624 [0.555 ; 0.686]	0.649 [0.591 ; 0.714]	0.593 [0.511 ; 0.667]
B. AUC	Null model*	0.708 [0.65 ; 0.761]	0.709 [0.652 ; 0.762]	0.717 [0.656 ; 0.771]	0.675 [0.609 ; 0.74]
	ORD*	0.73 [0.675 ; 0.779]	0.717 [0.66 ; 0.767]	0.727 [0.67 ; 0.783]	0.686 [0.616 ; 0.756]
	OVLT*	0.746 [0.698 ; 0.791]	0.73 [0.672 ; 0.778]	0.733 [0.674 ; 0.786]	0.692 [0.621 ; 0.755]
	EVLT*	0.744 [0.695 ; 0.791]	0.729 [0.672 ; 0.776]	0.732 [0.673 ; 0.784]	0.69 [0.619 ; 0.76]
	ESB*	0.723 [0.67 ; 0.773]	0.714 [0.655 ; 0.767]	0.728 [0.668 ; 0.777]	0.697 [0.636 ; 0.761]
	ESLT*	0.709 [0.652 ; 0.762]	0.7 [0.644 ; 0.752]	0.709 [0.648 ; 0.767]	0.671 [0.604 ; 0.74]
C. Brier	Null Model	0.193 [0.174 ; 0.212]	0.226 [0.212 ; 0.24]	0.234 [0.221 ; 0.247]	0.251 [0.235 ; 0.269]
	ORD	0.193 [0.174 ; 0.212]	0.227 [0.212 ; 0.241]	0.236 [0.223 ; 0.249]	0.25 [0.235 ; 0.268]
	OVLT	0.171 [0.151 ; 0.191]	0.204 [0.187 ; 0.224]	0.215 [0.199 ; 0.233]	0.232 [0.211 ; 0.254]
	EVLT	0.171 [0.151 ; 0.192]	0.204 [0.187 ; 0.224]	0.216 [0.199 ; 0.234]	0.233 [0.211 ; 0.254]
	ESB	0.188 [0.169 ; 0.207]	0.22 [0.205 ; 0.237]	0.227 [0.213 ; 0.245]	0.241 [0.222 ; 0.263]
	ESLT	0.193 [0.174 ; 0.212]	0.227 [0.212 ; 0.242]	0.236 [0.223 ; 0.25]	0.252 [0.235 ; 0.269]
D. Brier	Null model*	0.181 [0.161 ; 0.2]	0.208 [0.191 ; 0.228]	0.214 [0.196 ; 0.234]	0.229 [0.209 ; 0.251]
	ORD*	0.174 [0.153 ; 0.194]	0.204 [0.186 ; 0.225]	0.211 [0.193 ; 0.231]	0.227 [0.203 ; 0.25]
	OVLT*	0.169 [0.149 ; 0.188]	0.199 [0.182 ; 0.219]	0.208 [0.191 ; 0.227]	0.223 [0.202 ; 0.246]
	EVLT*	0.169 [0.148 ; 0.189]	0.199 [0.182 ; 0.219]	0.209 [0.192 ; 0.226]	0.224 [0.202 ; 0.247]
	ESB*	0.178 [0.159 ; 0.196]	0.207 [0.189 ; 0.227]	0.212 [0.194 ; 0.232]	0.223 [0.202 ; 0.245]
	ESLT*	0.18 [0.16 ; 0.198]	0.209 [0.192 ; 0.228]	0.217 [0.199 ; 0.236]	0.23 [0.209 ; 0.253]

Null model: survival model stratified on study, ORD: observed relative decline, OVLT: observed value at landmark time, EVLT: estimated value at landmark time, ESB: estimated slope at baseline, ESLT: Estimated slope at landmark time

* models with CA-125 at baseline

Figure S1: Calibration plot for the observed rate of decline (ORD) - 3 months

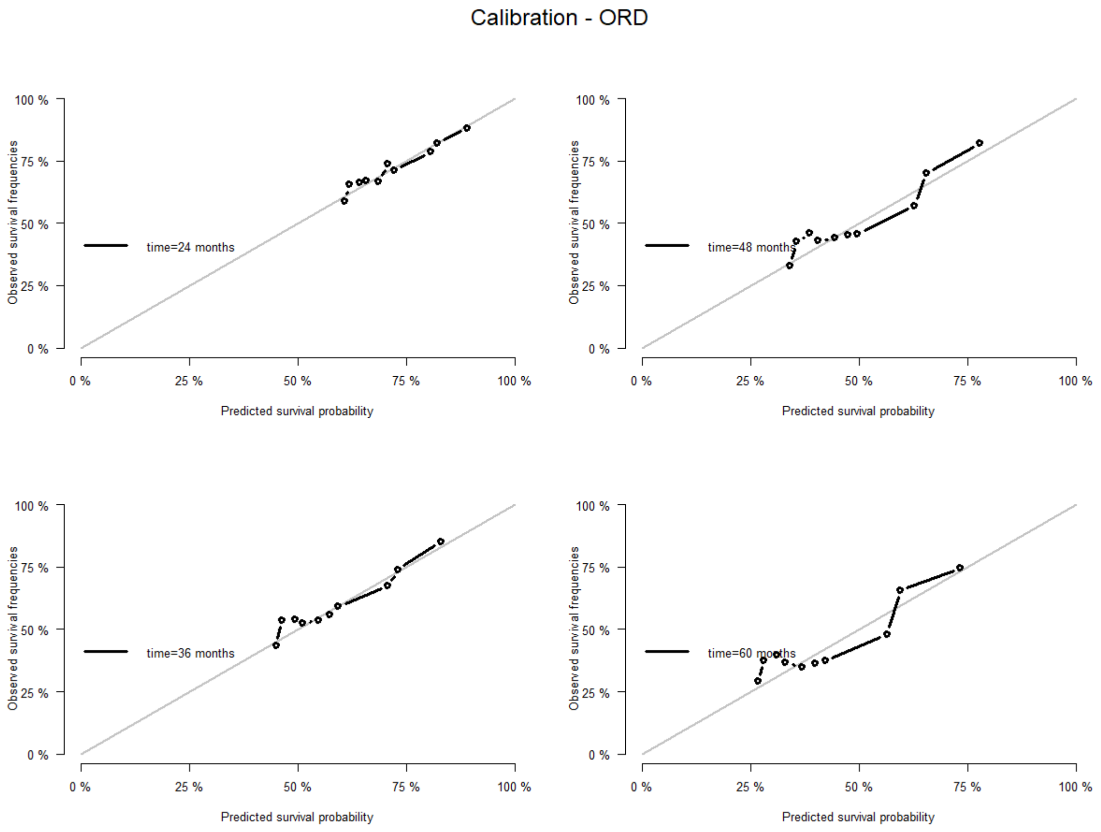

Figure S2: Calibration plot for the observed value at landmark time (OVLT) - 3 months

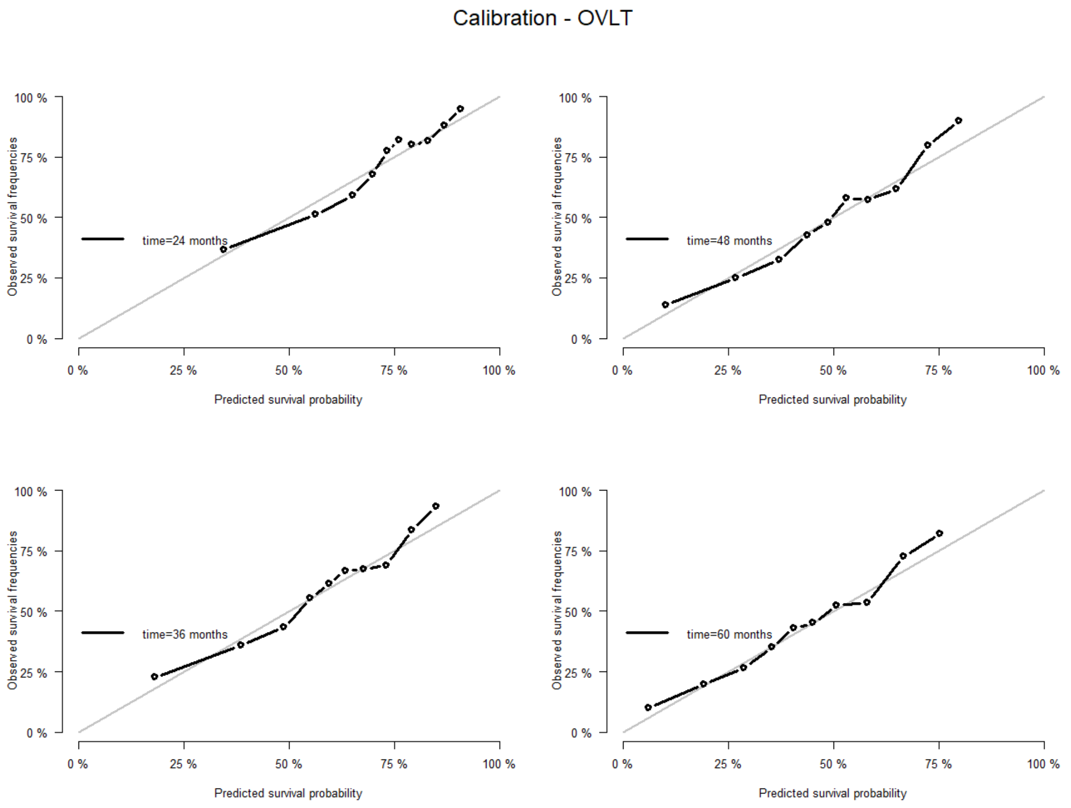

Figure S3: Calibration plot for the estimated value at landmark time (EVLT) - 3 months

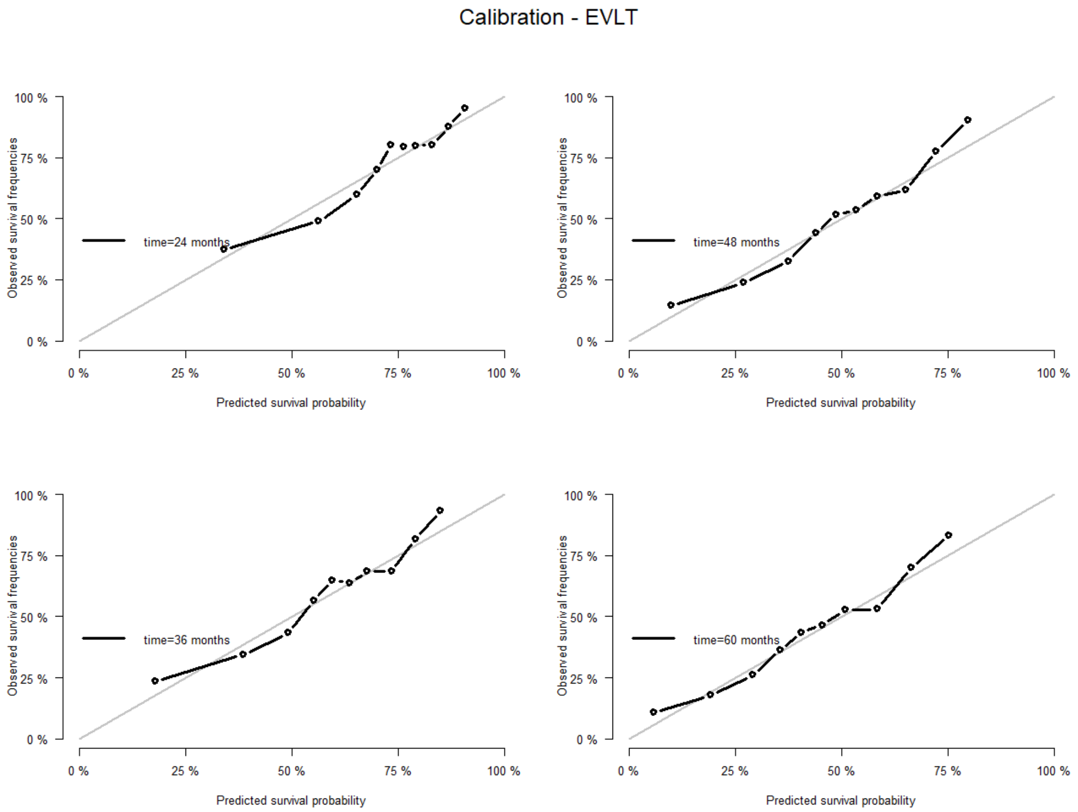

Figure S4: Calibration plot for the estimated slope at baseline (ESB) (for landmark 3 months)

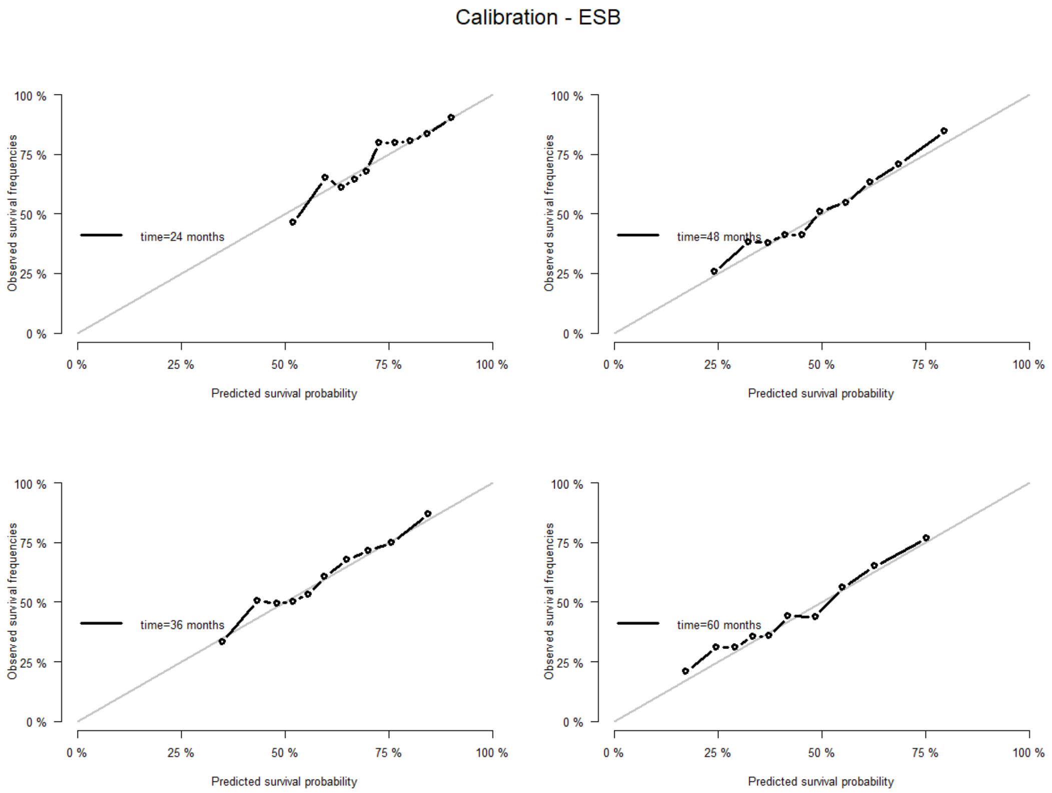

Figure S5: Calibration plot for the estimated slope at landmark time (ESLT)-3 months

Calibration - ESLT

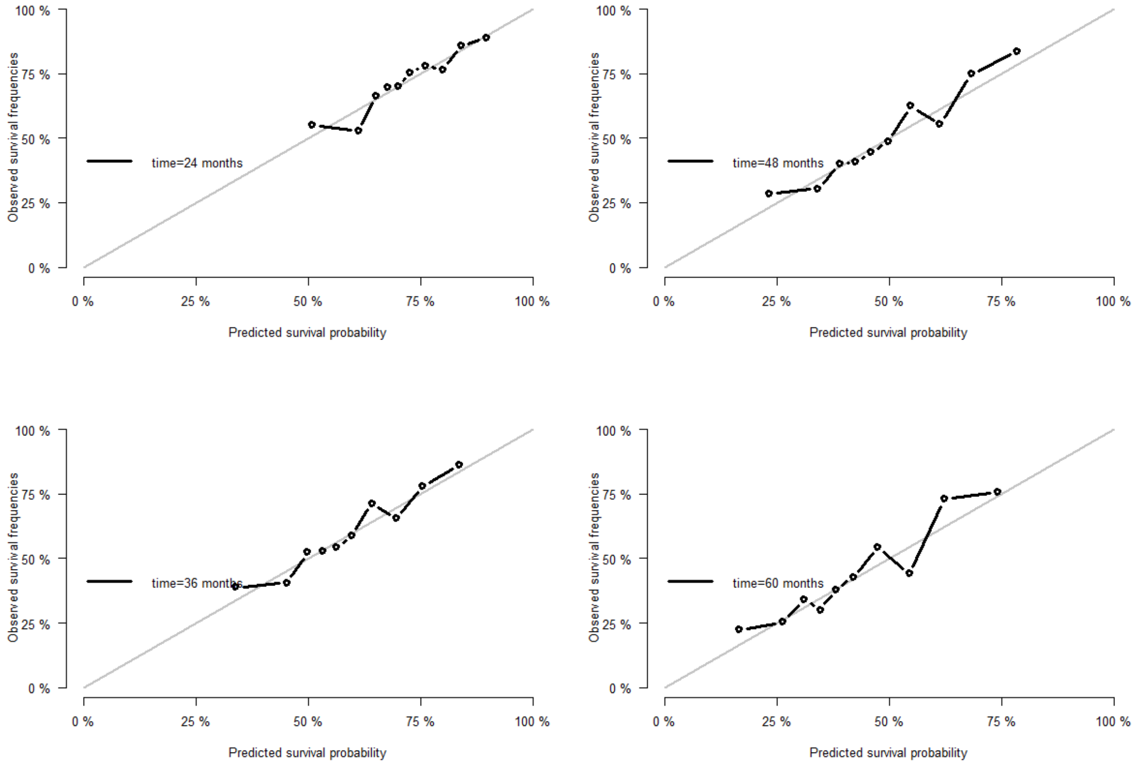

Table S4: Predictive performances by subgroups for estimated CA-125 value at 3 months

	Time	T=24	T=36	T=48	T=60
A. AUC	Figo I & II	0.684 [0.446 ; 0.878]	0.653 [0.467 ; 0.839]	0.683 [0.495 ; 0.861]	0.581 [0.338 ; 0.833]
	Figo III	0.709 [0.64 ; 0.772]	0.694 [0.621 ; 0.766]	0.714 [0.645 ; 0.78]	0.651 [0.569 ; 0.725]
	Figo IV	0.688 [0.563 ; 0.798]	0.692 [0.548 ; 0.816]	0.698 [0.524 ; 0.847]	0.69 [0.506 ; 0.861]
	Res Dis <1cm	0.687 [0.589 ; 0.79]	0.679 [0.577 ; 0.771]	0.698 [0.606 ; 0.788]	0.649 [0.553 ; 0.748]
	Res Dis >=1cm	0.707 [0.634 ; 0.768]	0.707 [0.629 ; 0.782]	0.72 [0.632 ; 0.796]	0.662 [0.548 ; 0.763]
B. Brier	Figo I & II	0.091 [0.062 ; 0.123]	0.132 [0.097 ; 0.168]	0.162 [0.124 ; 0.207]	0.204 [0.156 ; 0.258]
	Figo III	0.19 [0.167 ; 0.214]	0.221 [0.197 ; 0.246]	0.216 [0.196 ; 0.239]	0.231 [0.21 ; 0.254]
	Figo IV	0.235 [0.187 ; 0.286]	0.23 [0.189 ; 0.276]	0.201 [0.156 ; 0.241]	0.181 [0.142 ; 0.228]
	Res Dis <1cm	0.124 [0.099 ; 0.151]	0.175 [0.148 ; 0.2]	0.2 [0.175 ; 0.224]	0.23 [0.203 ; 0.256]
	Res Dis >=1cm	0.218 [0.193 ; 0.247]	0.224 [0.201 ; 0.249]	0.205 [0.182 ; 0.233]	0.211 [0.182 ; 0.243]

Figure S6 : Predictive performances measured by AUC (A) and Brier Score (B) by subgroups for estimated CA-125 value at 3 months

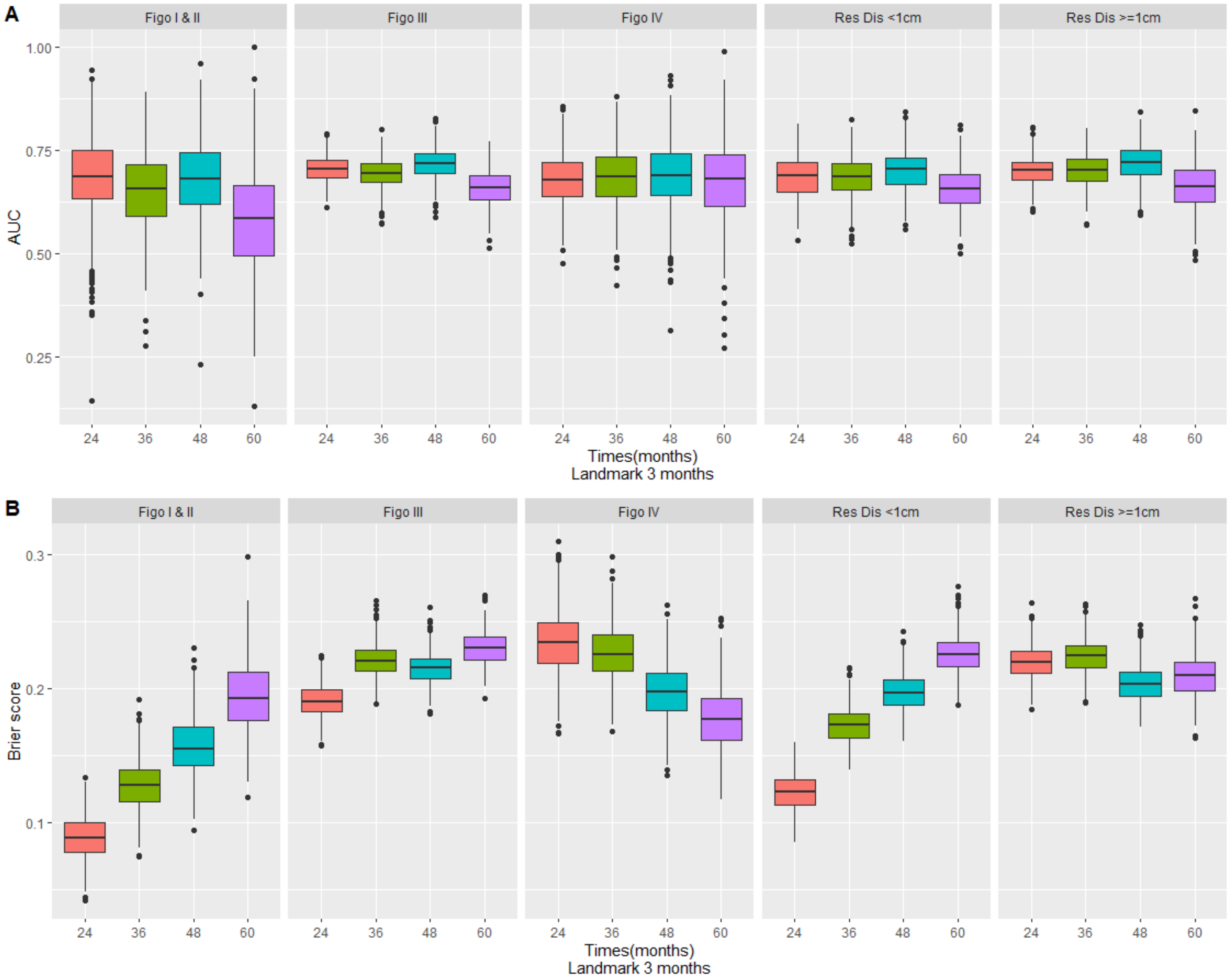

Table S5: Predictive performances by subgroups for estimated CA-125 value at 6 months

A. AUC	Time	T=24	T=36	T=48	T=60
	Figo I & II	0.649 [0.442 ; 0.85]	0.656 [0.435 ; 0.843]	0.665 [0.443 ; 0.85]	0.553 [0.311 ; 0.8]
	Figo III	0.7 [0.636 ; 0.767]	0.682 [0.599 ; 0.745]	0.681 [0.61 ; 0.758]	0.627 [0.53 ; 0.712]
	Figo IV	0.688 [0.55 ; 0.821]	0.678 [0.518 ; 0.818]	0.684 [0.499 ; 0.858]	0.653 [0.433 ; 0.877]
	Res Dis <1cm	0.682 [0.591 ; 0.771]	0.674 [0.587 ; 0.759]	0.683 [0.596 ; 0.765]	0.622 [0.519 ; 0.721]
	Res Dis >=1cm	0.704 [0.63 ; 0.775]	0.691 [0.592 ; 0.774]	0.687 [0.589 ; 0.777]	0.632 [0.509 ; 0.741]
B. Brier	Figo I & II	0.093 [0.063 ; 0.123]	0.134 [0.099 ; 0.172]	0.171 [0.13 ; 0.219]	0.223 [0.169 ; 0.282]
	Figo III	0.19 [0.166 ; 0.215]	0.222 [0.198 ; 0.246]	0.225 [0.203 ; 0.245]	0.237 [0.213 ; 0.263]
	Figo IV	0.233 [0.177 ; 0.295]	0.236 [0.191 ; 0.285]	0.212 [0.166 ; 0.252]	0.198 [0.148 ; 0.246]
	Res Dis <1cm	0.126 [0.103 ; 0.152]	0.177 [0.154 ; 0.203]	0.207 [0.184 ; 0.232]	0.241 [0.212 ; 0.272]
	Res Dis >=1cm	0.218 [0.192 ; 0.25]	0.229 [0.201 ; 0.26]	0.217 [0.191 ; 0.243]	0.219 [0.188 ; 0.251]

Figure S7 : Predictive performances measured by AUC (A) and Brier Score (B) by subgroups for estimated CA-125 value at 6 months

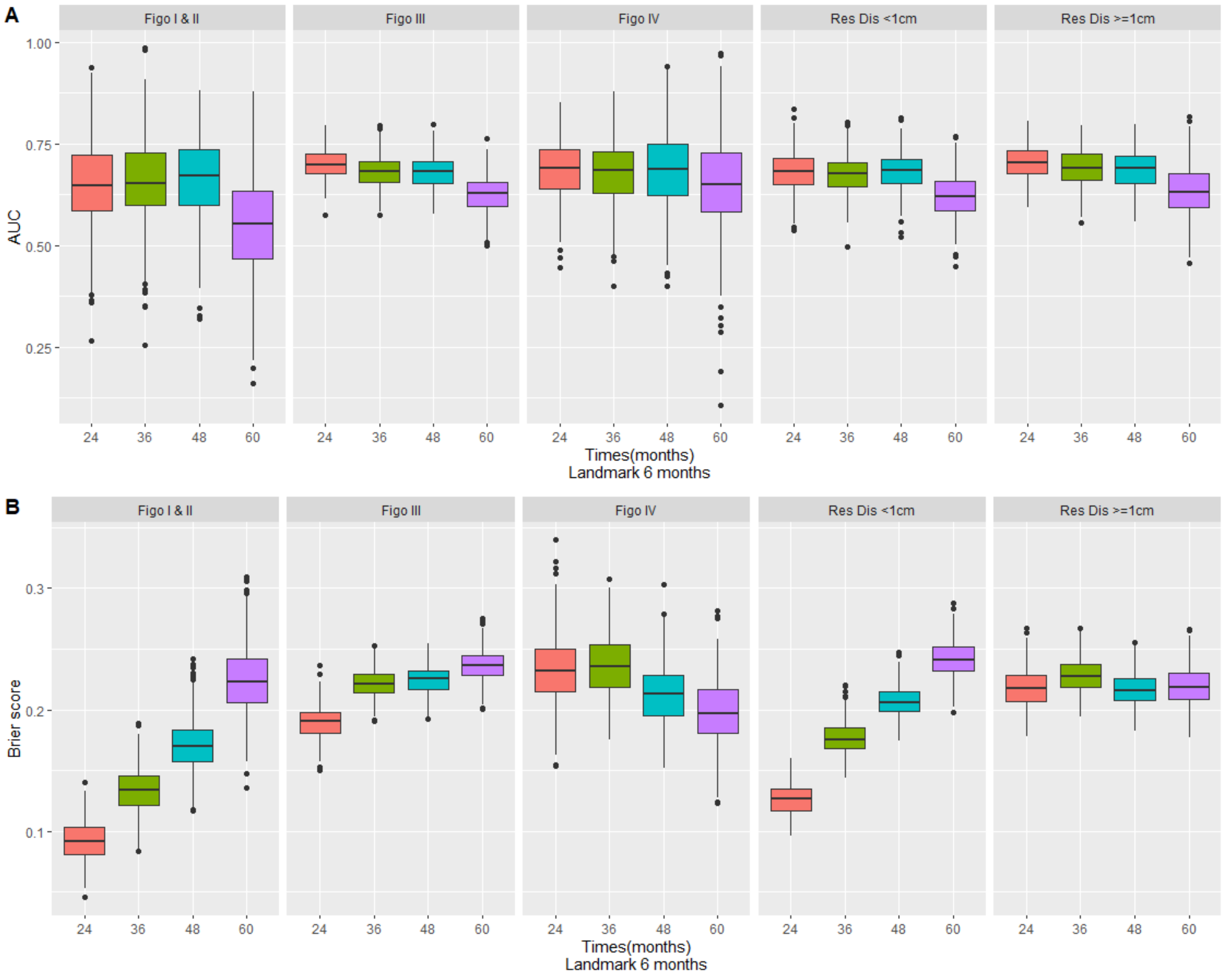

Table S6: 5-fold cross-validated AUC (A,B) and Brier score (C,D) for the prediction of overall survival at 24, 36, 48 and 60 months from CA125 history up to 3 months when considering different CA125 summaries. Half population including only Standard regimen arm

	Time	T=24	T=36	T=48	T=60
A. AUC	Null model	0.592 [0.51 ; 0.667]	0.6 [0.522 ; 0.667]	0.588 [0.512 ; 0.656]	0.536 [0.46 ; 0.603]
	ORD	0.585 [0.503 ; 0.672]	0.604 [0.53 ; 0.67]	0.592 [0.505 ; 0.671]	0.544 [0.461 ; 0.624]
	OVLT	0.714 [0.618 ; 0.793]	0.702 [0.613 ; 0.77]	0.7 [0.603 ; 0.769]	0.663 [0.575 ; 0.742]
	EVLT	0.712 [0.618 ; 0.791]	0.701 [0.616 ; 0.769]	0.701 [0.608 ; 0.773]	0.667 [0.584 ; 0.745]
	ESB	0.642 [0.548 ; 0.739]	0.636 [0.554 ; 0.706]	0.629 [0.548 ; 0.699]	0.582 [0.501 ; 0.657]
	ESLT	0.634 [0.547 ; 0.73]	0.645 [0.565 ; 0.716]	0.638 [0.558 ; 0.712]	0.595 [0.511 ; 0.671]
B. AUC	Null model*	0.67 [0.587 ; 0.752]	0.687 [0.608 ; 0.761]	0.691 [0.599 ; 0.777]	0.662 [0.584 ; 0.74]
	ORD*	0.695 [0.598 ; 0.779]	0.706 [0.621 ; 0.778]	0.715 [0.628 ; 0.792]	0.687 [0.608 ; 0.764]
	OVLT*	0.713 [0.619 ; 0.792]	0.713 [0.628 ; 0.782]	0.716 [0.625 ; 0.792]	0.685 [0.599 ; 0.762]
	EVLT*	0.711 [0.618 ; 0.793]	0.712 [0.629 ; 0.78]	0.716 [0.625 ; 0.792]	0.686 [0.604 ; 0.759]
	ESB*	0.683 [0.595 ; 0.765]	0.696 [0.612 ; 0.768]	0.699 [0.609 ; 0.774]	0.671 [0.592 ; 0.749]
	ESLT*	0.67 [0.587 ; 0.751]	0.686 [0.607 ; 0.761]	0.69 [0.596 ; 0.771]	0.661 [0.584 ; 0.741]
C. Brier	Null Model	0.162 [0.131 ; 0.196]	0.216 [0.193 ; 0.24]	0.243 [0.225 ; 0.265]	0.263 [0.246 ; 0.29]
	ORD	0.163 [0.132 ; 0.196]	0.217 [0.195 ; 0.24]	0.244 [0.225 ; 0.266]	0.263 [0.246 ; 0.289]
	OVLT	0.153 [0.124 ; 0.185]	0.197 [0.17 ; 0.229]	0.213 [0.19 ; 0.244]	0.232 [0.21 ; 0.262]
	EVLT	0.152 [0.124 ; 0.185]	0.197 [0.169 ; 0.229]	0.212 [0.189 ; 0.244]	0.231 [0.21 ; 0.259]
	ESB	0.16 [0.13 ; 0.193]	0.212 [0.188 ; 0.236]	0.236 [0.217 ; 0.261]	0.256 [0.236 ; 0.281]
	ESLT	0.161 [0.13 ; 0.194]	0.211 [0.187 ; 0.236]	0.235 [0.216 ; 0.258]	0.254 [0.233 ; 0.277]
D. Brier	Null model*	0.158 [0.127 ; 0.191]	0.203 [0.177 ; 0.229]	0.22 [0.194 ; 0.249]	0.235 [0.21 ; 0.265]
	ORD*	0.153 [0.126 ; 0.188]	0.196 [0.169 ; 0.224]	0.209 [0.184 ; 0.239]	0.226 [0.201 ; 0.253]
	OVLT*	0.153 [0.125 ; 0.187]	0.196 [0.168 ; 0.227]	0.21 [0.186 ; 0.242]	0.227 [0.204 ; 0.257]
	EVLT*	0.153 [0.125 ; 0.187]	0.195 [0.167 ; 0.226]	0.209 [0.185 ; 0.243]	0.226 [0.203 ; 0.255]
	ESB*	0.156 [0.126 ; 0.191]	0.201 [0.175 ; 0.227]	0.217 [0.193 ; 0.245]	0.233 [0.209 ; 0.261]
	ESLT*	0.158 [0.127 ; 0.191]	0.203 [0.177 ; 0.229]	0.22 [0.194 ; 0.25]	0.236 [0.21 ; 0.265]

Null model: survival model stratified on study, ORD: observed relative decline, OVLT: observed value at landmark time, EVLT: estimated value at landmark time, ESB: estimated slope at baseline, ESLT: estimated slope at landmark time

* models with CA-125 at baseline

Figure S8: 5-fold cross-validated AUC (A,B) and Brier score (C,D) for the prediction of overall survival at 24, 36, 48 and 60 months from CA125 history up to 3 months in Standard regimen arm when considering different CA125 summaries (ORD, OVLT, EVLT, ESB, ESLT) adjusted (B,D) or not (A,C) on the CA125 value at baseline. ** models with CA-125 at baseline*

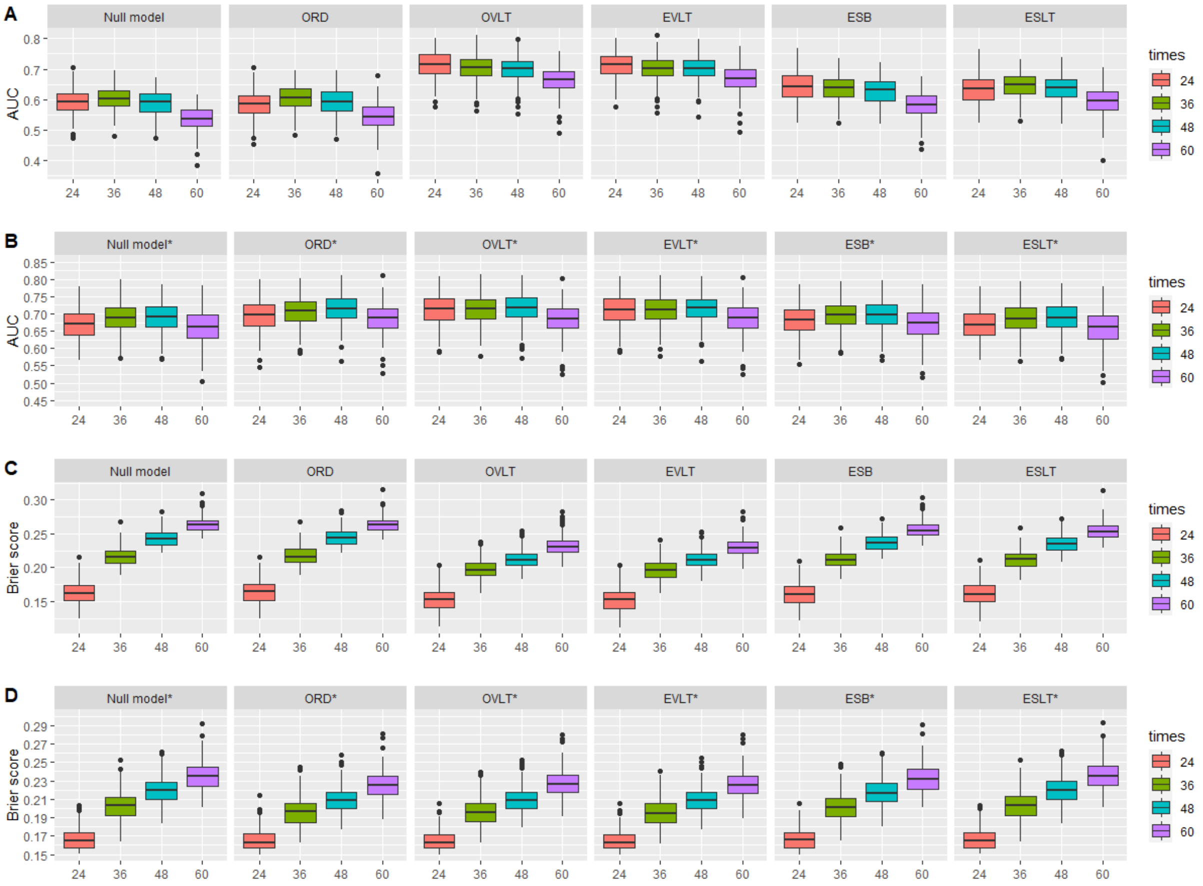

Table S7: 5-fold cross-validated AUC (A,B) and Brier score (C,D) for the prediction of overall survival at 24, 36, 48 and 60 months from CA125 history up to 6 months when considering different CA125 summaries. Half population including only Standard regimen arm

A. AUC	Time	T=24	T=36	T=48	T=60
	Null model	0.588 [0.53 ; 0.649]	0.589 [0.539 ; 0.644]	0.58 [0.532 ; 0.628]	0.534 [0.478 ; 0.587]
	ORD	0.595 [0.529 ; 0.664]	0.607 [0.549 ; 0.667]	0.604 [0.549 ; 0.654]	0.573 [0.515 ; 0.626]
	OVLT	0.727 [0.667 ; 0.777]	0.704 [0.65 ; 0.755]	0.686 [0.632 ; 0.735]	0.641 [0.58 ; 0.697]
	EVLT	0.721 [0.659 ; 0.774]	0.704 [0.649 ; 0.751]	0.687 [0.631 ; 0.737]	0.638 [0.572 ; 0.691]
	ESB	0.634 [0.575 ; 0.694]	0.641 [0.594 ; 0.695]	0.635 [0.578 ; 0.683]	0.598 [0.542 ; 0.66]
	ESLT	0.592 [0.532 ; 0.657]	0.598 [0.543 ; 0.651]	0.597 [0.541 ; 0.648]	0.562 [0.505 ; 0.619]
B. AUC	Null model*	0.683 [0.625 ; 0.738]	0.687 [0.632 ; 0.743]	0.683 [0.627 ; 0.732]	0.66 [0.603 ; 0.713]
	ORD*	0.705 [0.646 ; 0.757]	0.703 [0.646 ; 0.754]	0.702 [0.649 ; 0.752]	0.674 [0.621 ; 0.724]
	OVLT*	0.737 [0.677 ; 0.785]	0.727 [0.673 ; 0.777]	0.715 [0.657 ; 0.769]	0.685 [0.627 ; 0.733]
	EVLT*	0.733 [0.67 ; 0.782]	0.725 [0.667 ; 0.776]	0.715 [0.656 ; 0.771]	0.681 [0.623 ; 0.732]
	ESB*	0.696 [0.632 ; 0.752]	0.701 [0.645 ; 0.755]	0.697 [0.643 ; 0.748]	0.676 [0.619 ; 0.729]
	ESLT*	0.682 [0.621 ; 0.737]	0.686 [0.629 ; 0.743]	0.682 [0.627 ; 0.732]	0.659 [0.603 ; 0.714]
C. Brier	Null Model	0.167 [0.148 ; 0.187]	0.214 [0.198 ; 0.231]	0.24 [0.228 ; 0.255]	0.264 [0.25 ; 0.28]
	ORD	0.168 [0.149 ; 0.189]	0.215 [0.198 ; 0.232]	0.241 [0.227 ; 0.256]	0.263 [0.249 ; 0.279]
	OVLT	0.148 [0.129 ; 0.168]	0.191 [0.175 ; 0.214]	0.215 [0.2 ; 0.234]	0.238 [0.221 ; 0.258]
	EVLT	0.148 [0.129 ; 0.169]	0.191 [0.174 ; 0.213]	0.215 [0.199 ; 0.235]	0.239 [0.222 ; 0.259]
	ESB	0.164 [0.145 ; 0.184]	0.208 [0.192 ; 0.226]	0.232 [0.218 ; 0.249]	0.253 [0.235 ; 0.271]
	ESLT	0.167 [0.147 ; 0.187]	0.214 [0.197 ; 0.231]	0.239 [0.225 ; 0.254]	0.261 [0.247 ; 0.277]
D. Brier	Null model*	0.158 [0.142 ; 0.181]	0.199 [0.182 ; 0.217]	0.218 [0.201 ; 0.235]	0.235 [0.216 ; 0.253]
	ORD*	0.153 [0.136 ; 0.175]	0.194 [0.177 ; 0.215]	0.212 [0.196 ; 0.23]	0.231 [0.213 ; 0.25]
	OVLT*	0.147 [0.131 ; 0.168]	0.188 [0.171 ; 0.209]	0.208 [0.192 ; 0.227]	0.227 [0.209 ; 0.248]
	EVLT*	0.148 [0.131 ; 0.168]	0.188 [0.171 ; 0.209]	0.208 [0.191 ; 0.229]	0.228 [0.211 ; 0.248]
	ESB*	0.157 [0.141 ; 0.179]	0.196 [0.178 ; 0.216]	0.214 [0.196 ; 0.234]	0.23 [0.212 ; 0.248]
	ESLT*	0.159 [0.142 ; 0.181]	0.199 [0.182 ; 0.218]	0.219 [0.201 ; 0.236]	0.235 [0.216 ; 0.253]

Null model: survival model stratified on study, ORD: observed relative decline, OVLT: observed value at landmark time, EVLT: estimated value at landmark time, ESB: estimated slope at baseline, ESLT: estimated slope at landmark time

* models with CA-125 at baseline

Figure S9: 5-fold cross-validated AUC (A,B) and Brier score (C,D) for the prediction of overall survival at 24, 36, 48 and 60 months from CA125 history up to 6 months in Standard regimen arm when considering different CA125 summaries (ORD, OVLT, EVLT, ESB, ESLT) adjusted (B,D) or not (A,C) on the CA125 value at baseline. * models with CA-125 at baseline

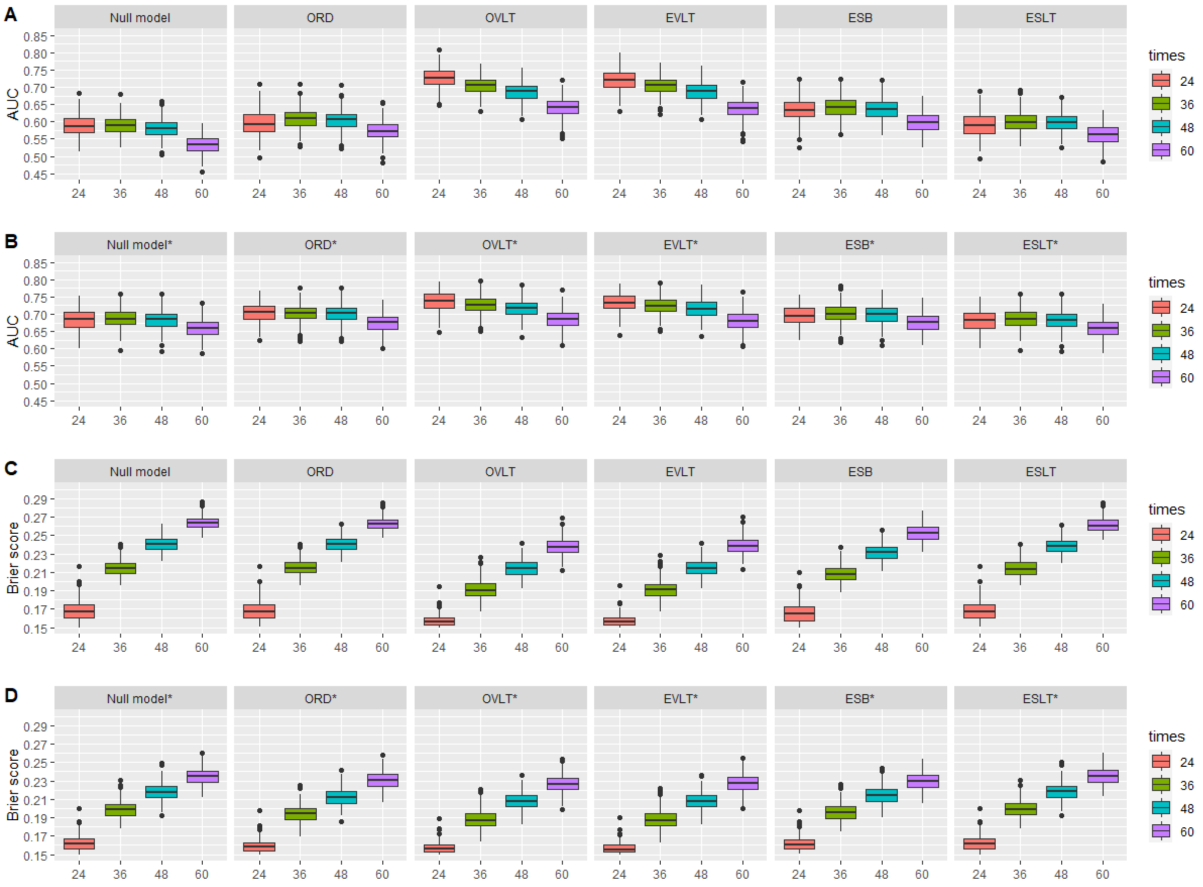

Supplement: Supplementary file 1 [file cancers-15-01823-s001.zip › cancers-2231670-supplementary.pdf]
